# MqsR toxin as a biotechnological tool for plant pathogen bacterial control

**DOI:** 10.1038/s41598-022-06690-x

**Published:** 2022-02-18

**Authors:** Reinaldo Rodrigues de Souza-Neto, Isis Gabriela Barbosa Carvalho, Paula Maria Moreira Martins, Simone Cristina Picchi, Juarez Pires Tomaz, Raquel Caserta, Marco Aurélio Takita, Alessandra Alves de Souza

**Affiliations:** 1 Citrus Research Center, Agronomic Institute - IAC, Cordeirópolis, SP Brazil; 2 Department of Genetics, Evolution, Microbiology, and Immunology, Institute of Biology, University of Campinas - UNICAMP, Campinas, SP Brazil; 3 Rural Development Institute of Parana - IAPAR-EMATER, Londrina, PR Brazil

**Keywords:** Molecular engineering in plants, Antimicrobials

## Abstract

Type II toxin-antitoxin (TA) systems are widespread in bacteria and are involved in important cell features, such as cell growth inhibition and antimicrobial tolerance, through the induction of persister cells. Overall, these characteristics are associated with bacterial survival under stress conditions and represent a significant genetic mechanism to be explored for antibacterial molecules. We verified that even though *Xylella fastidiosa* and *Xanthomonas citri* subsp. *citri* share closely related genomes, they have different Type II TA system contents. One important difference is the absence of *mqsRA* in *X. citri*. The toxin component of this TA system has been shown to inhibit the growth of *X. fastidiosa*. Thus, the absence of *mqsRA* in *X. citri* led us to explore the possibility of using the MqsR toxin to impair *X. citri* growth. We purified MqsR and confirmed that the toxin was able to inhibit *X. citri*. Subsequently, transgenic citrus plants producing MqsR showed a significant reduction in citrus canker and citrus variegated chlorosis symptoms caused, respectively, by *X. citri* and *X. fastidiosa*. This study demonstrates that the use of toxins from TA systems is a promising strategy to be explored aiming bacterial control.

## Introduction

To feed a population of 9.7 billion by 2050, food production will need to increase from its current amount of 8.4 billion tons to almost 13.5 billion tons per year^1^. Under this scenario, technological innovation has been indicated to be a driver to help agricultural production and to ensure food security^2^. Additionally, crop losses due to pathogen attack are considered an important issue since it necessitates the use of large amounts of pesticides to maintain agricultural productivity; however, their use causes environmental damage and directly impacts agricultural sustainability.

Citrus is one of the most widely developed and economically important crops in the world^3^. However, bacterial diseases such as Huanglongbing (HLB), citrus canker and citrus variegated chlorosis (CVC) cause significant economic losses due to the decreases in fruit yield and increasing requirements of costly disease-control. Although agrochemicals are necessary to maintain fruit production, their application is often not sufficient to control bacterial diseases or even to prevent their spreading^4^. In addition, many of these products are toxic to the environment^5^. These facts indicate the necessity of alternatives to control plant diseases in a more sustainable way. Among these diseases, citrus canker and CVC, caused by *X. citri* and *X. fastidiosa*, respectively, are constantly treated with agrochemicals to limit the citrus production economic losses that they cause^6,7^. The genomes of both bacteria were sequenced^8,9^, and analyses revealed that they share high content similarity^10^. In addition, it was recently shown that *X. fastidiosa* and *X. citri* have many genes associated with type II toxin-antitoxin (TA) systems^11–16^.

The interest in bacterial TA systems is due to their functions in many important processes, such as cell growth inhibition, bacterial programmed cell death, anti-addiction (for maintenance of the plasmid in the cell), antibiotic tolerance through the induction of persister cells, biofilm formation and gene expression regulation^17–19^. Type II TA systems consist of a pair of genes in the same operon, which form a stable toxin and an unstable antitoxin that, under normal conditions, binds to the toxin and blocks its activity. However, the antitoxin is degraded by proteases under stress, allowing the toxin to act on specific targets in the cell^20^. Overall, many of these toxins act as ribonucleases and degrade the mRNA of target genes, which in turn decreases cell growth and metabolism. When normal growth conditions are re-established, the antitoxin is translated again through specific genetic regulation, and it blocks the toxin, allowing the cell to grow^20^. Thus, this mechanism represents an important bacterial strategy of survival under stress conditions, and for this reason, it has been studied in bacteria that cause chronic human diseases^21^. Recently, TA systems have also been investigated in bacteria that cause plant diseases^12,14,22,23^ and, in *X. fastidiosa*, this type of system has been demonstrated to be involved in bacterial adaptation, pathogenicity, biofilm formation, bacterial motility and gene expression regulation^11,12,14,16,24^.

It has been suggested that Type II TA systems are good candidates for developing novel antimicrobials due to the essential processes they target^25,26^. Following this approach, we verified that even though *X. citri* and *X. fastidiosa* have closely related genomes, each has specific Type II TA systems^15,22^. Among them, the *mqsRA* system is present in *X. fastidiosa* and absent in *X. citri*^22^. The *mqsRA* system was reported to be involved in biofilm formation and to induce persister cells in *Escherichia coli*^19^. This system is composed of the MqsR toxin, an RNase that cleaves mRNA at GCU sites^27,28^ and the MqsA antitoxin, which binds to the toxin via its N-terminal domain and to DNA via the helix-turn-helix (HTH) motif in its C-terminal domain^27^. The *X. fastidiosa* MqsR was also demonstrated to cleave mRNA primarily at GCU sites, and its MqsA was shown to inhibit the toxin by direct binding^12,14,16^. Additionally, in *X. fastidiosa*, MqsR is involved in biofilm formation, growth inhibition and persister cell induction under stress conditions^12,14,16^. Therefore, as *X. citri* does not have MqsRA, we investigated whether MqsR from *X. fastidiosa* could inhibit *X. citri* growth and consequently decrease disease symptoms in transgenic lines overexpressing this toxin. In addition, we evaluated if the overexpression of MqsR could also interfere in *X. fastidiosa* pathogenicity. In this study, we showed that MqsR was able to disturb *X. citri* cell growth. In addition, citrus transgenic lines overexpressing MqsR exhibited a significant reduction of citrus canker and citrus variegated chlorosis symptoms. These results highlight the potential of TA systems to be used as new biotechnology tools to control pathogenic bacteria.

## Results

### Purified MqsR toxin disturbs *X. citri* growth

**Figure 1:**
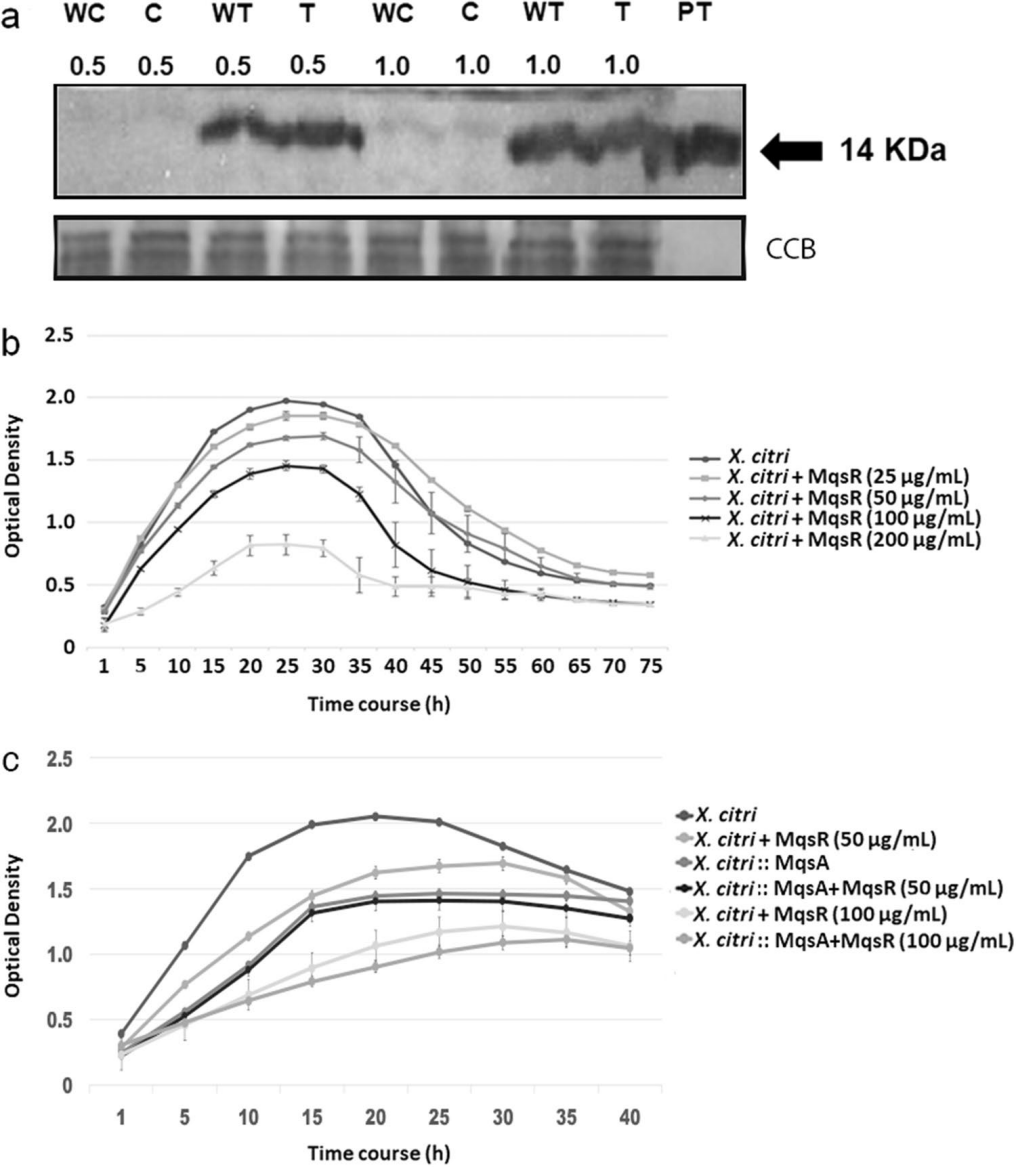
In vitro analysis of purified MqsR in *X. citri*. (**a**) Immunoblot detection of MqsR associated with *X. citri* cells. Four treatments were examined: WC – washed *X. citri* control; C—non-washed *X. citri* control; WT—washed *X. citri* treated with MqsR; and T—non-washed *X. citri* treated with MqsR. PT is purified MqsR (positive control). The values 0.5 and 1.0 correspond to the initial OD used, and 14 kDa is the expected size of MqsR with a His(6)-tag. Equivalent proteins loading was verified with Coomassie Brilliant Blue (CBB). In this case, two gels were run in parallel, where one was stained with Coomassie blue to show the concentration of proteins (Supplementary Fig. S1) and the other was used for western blot. (**b**) Growth curve of *X. citri* in the presence of MqsR. Five treatments were evaluated, *X. citri* treated with 25, 50, 100 and 200 µg/mL of MqsR and the non-treated control. After 10 h, the concentrations of 50, 100 and 200 µg mL^-1^ showed significantly lower growth than the non-treated control (*P* < 0.05). Full-length gels and blots are included in Supplemental information. (**c**) Growth curve of *X. citri* and *X. citri* overexpressing MqsA (*X. citri* :: MqsA) in the presence of 50 and 100 µg/mL of MqsR and the non-treated control. After 10 h, only the concentration of 100 µg mL^-1^ showed significantly lower growth in *X. citri* :: MqsA than the non-treated control (*P* < 0.05).

Aiming to use this toxin as an antimicrobial against *X. citri*, we first investigated whether heterologously expressed MqsR from *X. fastidiosa* was able to affect *X. citri*. First, we confirmed that *X. citri* RNAs were cleaved upon external treatment by MqsR (Fig. S1A). In addition, we verified that toxin targeted *X. citri* cells in two ways: (1) direct lysis after centrifugation with no washing and (2) three washes prior to lysis to remove any toxin possibly attached to the cell membrane. Indication that MqsR toxin targeted *X. citri* cells was observed in SDS-PAGE (Fig. S1B), which was confirmed by Western blot (Fig. 1a). No MqsR homologue is present in *X. citri* cells, as no positive signal was observed at both concentrations of cells in the mock controls (Fig. 1a). Conversely, a strong signal was observed when 100 µg/mL of toxin was added to the cells from both treatments (washed and non-washed) at both cell densities (OD_600_ 0.5 and 1.0) (Fig. 1a).

We also investigated the *X. citri* growth in presence of four concentrations of MqsR from *X. fastidiosa* (25, 50, 100, and 200 µg/mL). A significant growth reduction was observed after 10 h of incubation for 50 µg/mL and above (Fig. 1b). After 30 h the bacteria reach the death phase of bacterial growth curve. To show that this effect is unequivocally related to the MqsR toxin, another experiment was performed in which *X. citri* expressing MqsA was used for testing its capacity in blocking the lethal effect of its counterpart in vitro. Indeed, when using 50 µg/mL of toxin, the antitoxin was able to rescue the cells while 100 µg/mL of toxin led the cells to death, probably because the toxin amount was higher than the antitoxin could afford (Fig. 1c). However, the lethal effect of 100 µg/mL of toxin was not as high as the observed for the wild type. In addition, it is observed that the overexpression of MqsA by itself affect cell growth. Overall, the results demonstrated that MqsR from *X. fastidiosa* showed an inhibitory effect on *X. citri* growth; moreover, the effect was shown to be dose-dependent.

### Overexpression of the mqsR gene in sweet orange and Carrizo citrange transgenic lines

**Figure 2:**
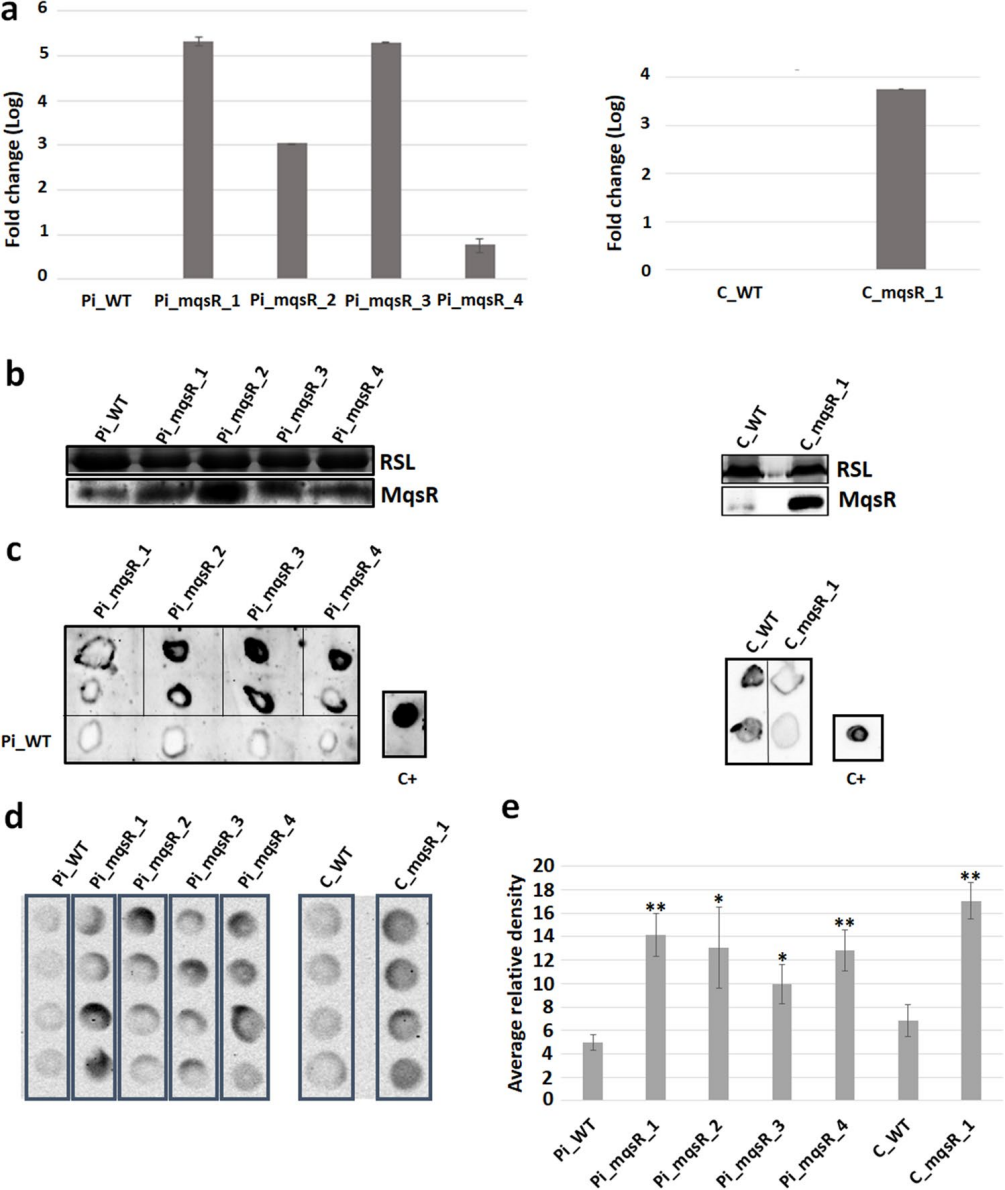
Molecular analyses of the transgenic lines. (**a**) Relative expression of *mqsR* in transgenic lines*.* Pi_WT, wild-type Pineapple sweet orange; Pi_mqsR_1, 2, 3, and 4, Pineapple transgenic lines transformed with *mqsR*; C_WT, wild-type Carrizo; and C_mqsR_1, Carrizo transgenic line transformed with *mqsR*. Expression was quantified in relation to the cyclophilin gene (endogenous control). (**b**) Detection of MqsR in Pineapple and Carrizo transgenic lines by Western blot analysis. Equivalent protein loading was verified with Coomassie Brilliant Blue, RLS corresponds to the ribulose-1,5-bisphosphate carboxylase large subunit. (**c**) Tissue-print of the transgenic lines. (**d**) Dot blot of transgenic lines. Pi_WT, wild-type Pineapple sweet orange; Pi_mqsR_1, 2, 3, and 4, Pineapple transgenic lines; C_WT, wild-type Carrizo; and C_mqsR_1, Carrizo transgenic line*.* C+ , purified MqsR (positive control). Full-length gels and blots are included in Supplemental information. (**e**) Quantification of dot blot. Quantification of relative unit of luminescence in the same area using Image Lab (Bio-Rad).

The *mqsR* gene from *X. fastidiosa* was successfully introduced into different citrus genotypes by *Agrobacterium tumefaciens* using the pCambia2301 vector with *mqsR* fused to a signal peptide to direct the toxin to the outside of the cell^29^. A total of ten Pineapple sweet orange (*Citrus sinensis*) and four Carrizo citrange (*C. sinensis* × *Poncirus trifoliata)* transgenic lines were confirmed by PCR (Fig. S2A). These lines were screened for symptom development to determine their susceptibility to *X. citri* after leaf infiltration. Four Pineapple sweet orange (Pi_mqsR_1, 2, 3 and 4) and one Carrizo citrange (C_mqsR_1) transgenic lines exhibited reduced citrus canker symptoms compared to wild type and other transgenic lines (Fig. S2B). Buds from these mother plants were then used for grafting onto Rangpur lime rootstocks to enable subsequent studies. The *mqsR* expression in the selected transgenic lines was confirmed by quantitative reverse-transcriptase (qRT)-PCR (Fig. 2a). In addition, the presence of MqsR was confirmed by Western Blot (Fig. 2b). Even though an unspecific band was detected in wild-type plants, the intensity of the bands was higher in transgenic lines. Thus, using other immunoblotting techniques, tissue-print and dot blot, we were able to confirm that these plants transcribe and translate *mqsR* (Fig. 2c, d, e).

Due to variation in transgene expression, we estimated the copy number of the *mqsR* for each transgenic lines to verify if different levels of transgene expression are related with number of inserts of *mqsR*. All transgenic lines showed one transgene copy per genome (Supplementary Table S1). Those data indicated that the difference in *mqsR* expression among the lines is probably related to the position where the insertion occurred in the host genome that can influence the expression of the transgene^30^, as also observed by Caserta and colleagues^31^.

### Citrus transgenic lines overexpressing MqsR have reduced citrus canker symptoms in detached leaves

**Figure 3:**
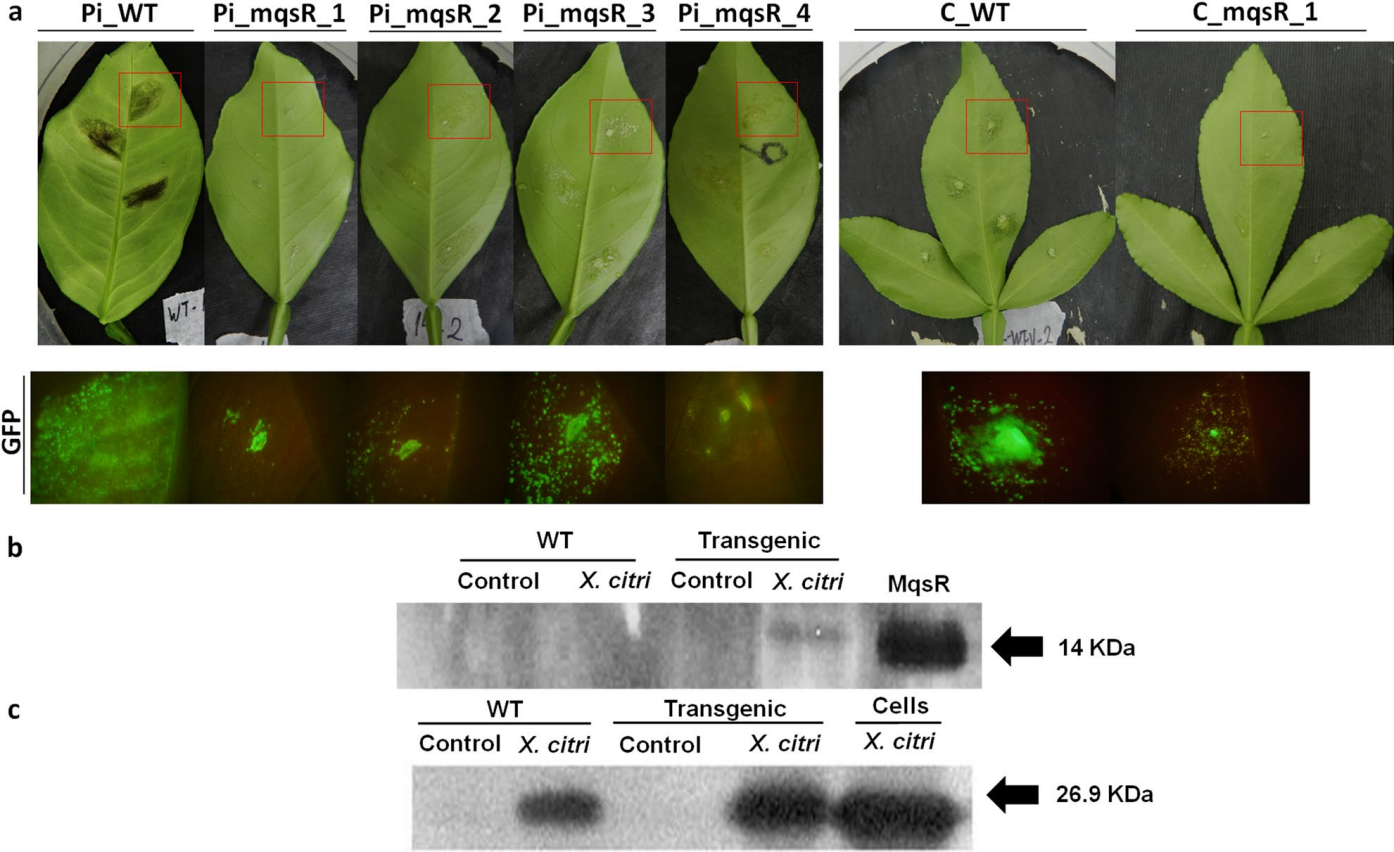
Citrus canker symptoms and MqsR detection in *X. citri*_GFP. (**a**) Citrus canker symptoms were evaluated at 14 DAI in wild-type and transgenic lines (top). The red squares correspond to the same area visualized by fluorescence microscopy (bottom). Green labelling indicates *X. citri*_GFP in the leaves. Pi_WT, wild-type Pineapple sweet orange; Pi_mqsR_1, 2, 3 and 4, transgenic Pineapple sweet orange lines; C_WT, wild-type Carrizo; and C_mqsR, transgenic Carrizo. (**b**) Detection of MqsR in *X. citri_GFP* cells by immunoblotting. The bacteria used in this experiment were isolated from the following plant lines: WT, wild-type; Transgenic (Pi_mqsR_1) representative line; leaf infiltrated with PBS (control); and, leaf infiltrated with *X. citri_GFP* (*X. citri*). MqsR corresponds to purified protein (positive control) for which the expected size is 14 kDa with a His(6)-tag. (**c**) Cells correspond to total proteins from *X. citri_GFP* (positive control). The expected size of GFP is 26.9 kDa. Full-length gels and blots are included in Supplemental information.

The observation that MqsR restricts growth of *X. citri* cells led us to investigate whether transgenic lines overexpressing *mqsR* could also affect symptom development. To test these hypotheses, we infiltrated leaves of transgenic and wild-type Pineapple sweet orange and Carrizo citrange plants with GFP-labelled *X. citri* (*X. citri*-GFP) and subsequently evaluated symptom development. Symptoms in leaves were assessed at 7 and 14 days after inoculation (DAI). No significant differences between transgenic and wild-type plants were observed at 7 DAI; however, at 14 DAI, typical symptoms of citrus canker, including hyperplasia, erumpent pustules, necrosis, and abscission of the petiole, were more evident in the wild-type plants compared to the transgenic lines (Fig. 3a). We also evaluated the *X. citri* population in both Pineapple sweet orange and Carrizo citrange transgenic lines which presented lower bacterial populations than those of the wild-type plants (Fig. S3).

The observation that symptoms were reduced in transgenic lines suggests that MqsR produced by the plant was affecting *X. citri,* as observed in vitro (Fig. 1). Thus, to confirm this hypothesis, the bacteria grown on transgenic and wild-type leaves were collected and used for immunoblotting analysis. Antibody reaction was observed only in *X. citri* from the transgenic leaves (Fig. 3b), indicated by a band corresponding to the size of MqsR (Fig. 3b). In addition, we confirmed our findings (Fig. 1a) that a homologous toxin is not present in *X. citri*, as the antibody against GFP was able to detect *X. citri*-GFP in both the wild-type and transgenic lines (Fig. 3c), but MqsR was detected only in *X. citri* in transgenic leaves (Fig. 3c). Therefore, these results suggest that MqsR from transgenic lines was able to affect *X. citri* and decreased canker development.

### Plants expressing mqsR show decreased citrus canker symptoms development

Since detached transgenic leaves showed increased resistance to *X. citri*, we investigated if such response could also occur in entire plants. Therefore, five plants of each line were propagated and sprayed with a suspension of *X. citri* and the severity of symptoms was assessed weekly using a diagrammatic scale^32^.

**Figure 4:**
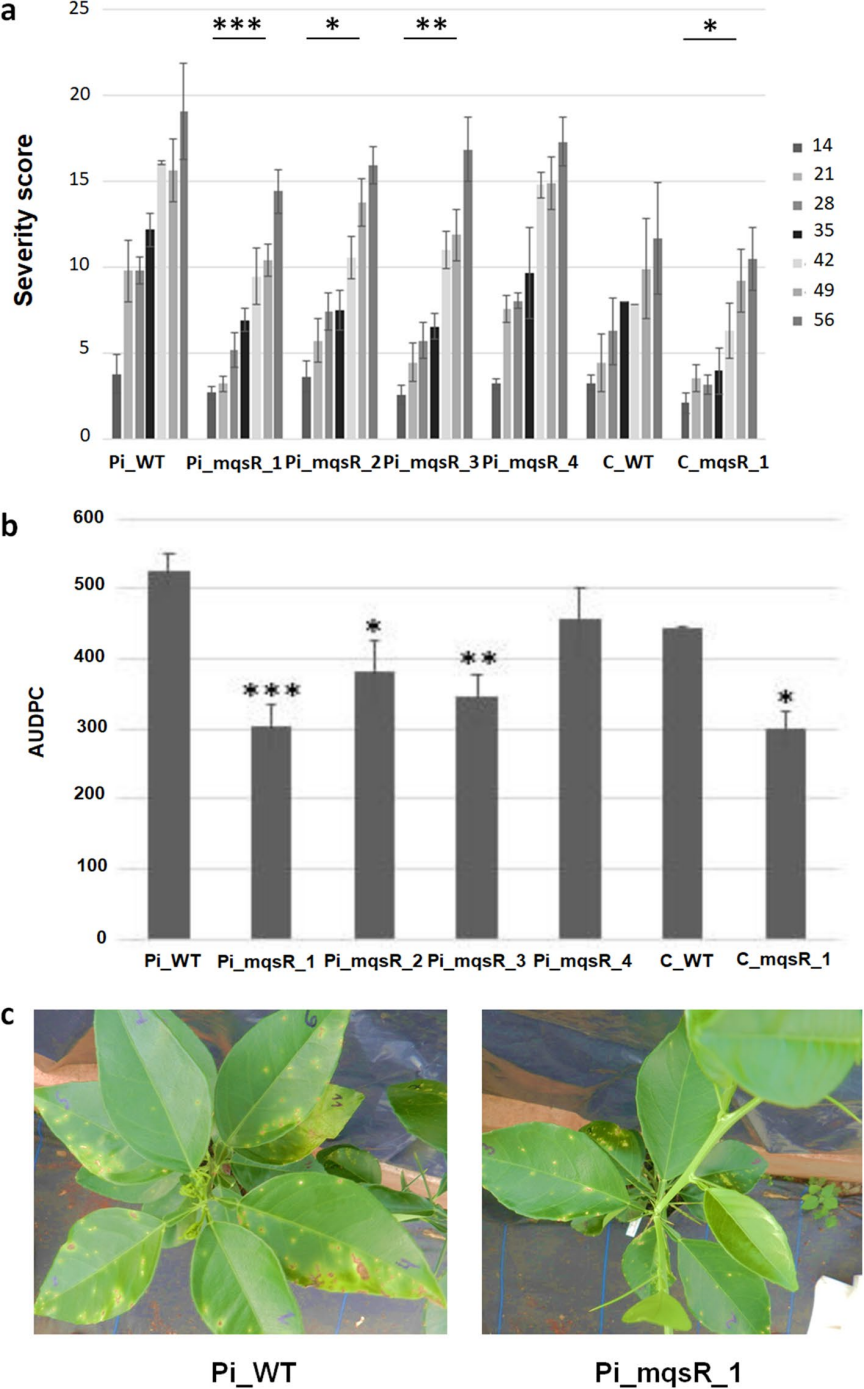
Citrus canker symptoms in whole plants of wild-type and transgenic lines. (**a**) Severity analysis of citrus canker symptoms were scored for five replicates of each transgenic line by three different evaluators at 14, 21, 28, 35, 42, 49, and 56 DAI. (**b**) Area under the disease progress curve (AUDPC) for citrus canker severity in wild-type and transgenic lines. The average of the citrus canker scores during disease development were used to calculate the disease progression curve. Asterisks show statistically significant differences for transgenic lines compared with their respective wild-type varieties using *t*-Student (*P < 0.05, ** *P* < 0.01, ***P < 0.001). Error bars represent the standard error of the means. Pi_WT, wild-type Pineapple sweet orange type; Pi_mqsR_1, 2, 3 and 4, Pineapple transgenic lines transformed with *mqsR*; C_WT, wild-type Carrizo; and C_mqsR_1, Carrizo transgenic line transformed with *mqsR*. (**c**) Severity of citrus canker symptoms in a representative wild-type Pineapple sweet orange plant (WT) and a transgenic Pineapple line (Transgenic).

All transgenic lines showed significant reduction of disease severity at all of the evaluated time points, with the exception of Pi_mqsR_4 (Fig. 4a). The difference in severity was more evident in the first points of the time course, 14, 21, 28 and 35 days after inoculation (Fig. 4a). To analyse the progress of disease as a function of time in the transgenic and wild-type plants, the symptom severity data were used to calculate the area under the disease progress curve (AUDPC). The results of the AUDPC analysis evidenced that all transgenic lines, except for Pi_mqsR_4, had significantly lower AUDPCs compared to those of the WT plants (Fig. 4b), indicating that the severity of disease in the transgenic lines Pi_mqsR_1, 2, and 3 and Ca_mqsR_1 was reduced throughout the disease progress. A representative picture of these symptoms, with a comparison of wild-type and transgenic lines, is shown in Fig. 4c.

### Plants expressing mqsR show decreased CVC symptoms development

In order to verify if these transgenic lines were producing a high amount of toxin that could be enough to affect even bacteria that present the MqsA antoxin, we used *X. fastidiosa* to test the model. Therefore, five plants of each sweet orange line were propagated and infected with *X. fastidiosa* and the severity of symptoms was assessed monthly using a diagrammatic scale^33^.

**Figure 5:**
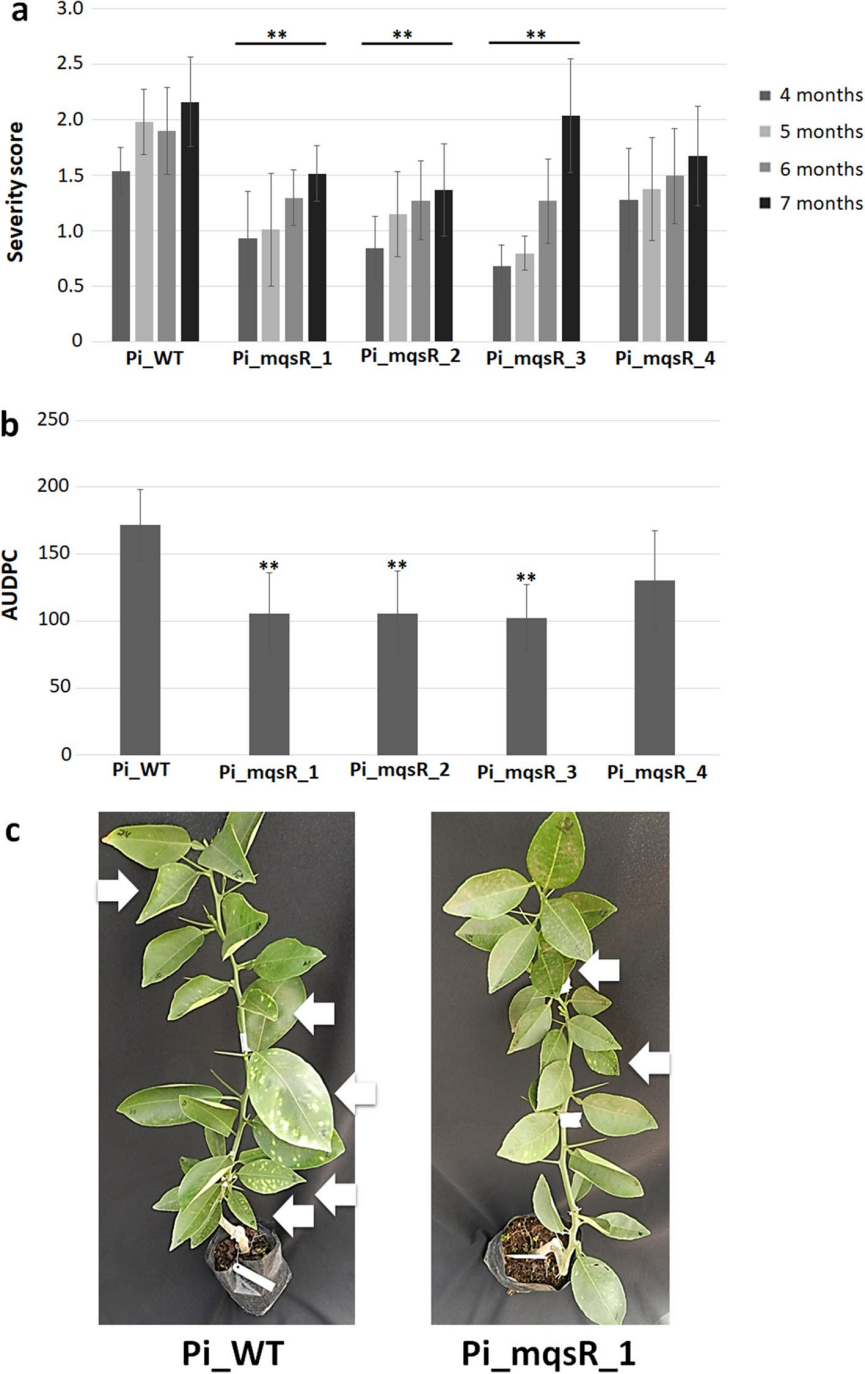
CVC symptoms in wild-type and transgenic lines. (**a**) Severity analysis of CVC symptoms. Plants were evaluated for at least five replicates of each transgenic line by three different evaluators at 4, 5, 6 and 7 months after inoculation. (**b**) Area under the disease progress curve (AUDPC) for CVC severity in wild-type and transgenic lines. The average of the CVC scores were used to calculate the disease progression curve. Asterisks show statistically significant differences for transgenic lines compared with wild-type using *t*-Student (*P < 0.05, ** *P* < 0.01, ***P < 0.001). Error bars represent the standard error of the means. Pi_WT, wild-type Pineapple sweet orange type; Pi_mqsR_1, 2, 3 and 4, Pineapple transgenic lines transformed with *mqsR*; C_WT. c) Illustration of the CVC symptoms showing the severity level on the leaves (white arrows) of wild-type (Pi_WT) and transgenic representative line (Pi_mqsR_1).

The events Pi_mqsR_1, 2, and 3 showed significantly lower severity levels compared to Pi_WT and Pi_mqsR_4 (Fig. 5a) during the time course of the experiment (Fig. 5a). These data were also used to calculate AUDPC to compare the disease progress over time with Pi_WT. The results from all transgenic lines, with exception Pi_mqsR_4, were significatively lower than wild type (Fig. 5b), demonstrating a delay in CVC symptoms development during the progress of disease. A representative picture comparing symptoms in wild type and transgenic lines is shown in Fig. 5c.

## Discussion

Citrus canker caused by *X. citri* is one of the most important citrus diseases as it affects all *Citrus* varieties. Since *Citrus* does not naturally present resistance genes to *X. citri*, different strategies have been developed to obtain plants that are resistant to this bacterium^7,34,35^. Transgenic technology is a powerful tool that can be used to improve food crop yields and decrease the use of agrochemicals; indeed, the use of this technology has consistently increased in the field over the years^36^.

Genetic transformation using genes from pathogens is an approach that has been successfully used to obtain plants resistant to *X. citri*^34,35^, *X. fastidiosa*^31,37^ and other phytopathogenic bacteria^38,39^. Although the toxin-antitoxin system has been highlighted as a promising strategy to decrease bacterial populations^11,14^, no study has ever explored this system as a biotechnological tool for phytopatogenic bacteria control. Thus, to use this strategy, we first verified that the MqsR toxin from *X. fastidiosa* affected *X. citri* growth in vitro (Fig. 1a). Even though different strategies were used, these results corroborate those already shown for *E. coli* and *X. fastidiosa,* i.e., the overexpression of *mqsR* reduced the growth rate compared to the wild-type bacterium^14,40,41^. The results show that a toxin from one phytobacterium can interfere with the growth of another bacterial species, possibly due to repression of MqsR target genes^41–43^ (Figure S4).

*X. citri* was sprayed on whole plants and most of the transgenic lines were more tolerant to citrus canker disease, with only exception, the transgenic line Pi_mqsR_4; although this line showed reduced symptoms in detached leaves compared to WT plants (Fig. 3a), it did not show statistically significant differences in symptom severity and AUDPC (Fig. 4). These findings could be explained by low levels of MqsR since Pi_mqsR_4 showed lower expression levels of *mqsR* compared to the other lines (Fig. 2b). Previous work shows that *X. fastidiosa* overexpressing *mqsR* was unable to induce symptoms in sweet orange after 2 years post inoculation^14^. Thus, to verify if overexpression of MqsR in transgenic lines could also interferes on CVC symptoms, we challenged these plants with *X. fastidiosa*. Even though in this study the symptoms were present in all transgenic lines, a significant lower disease progress over time was observed, with exception of Pi_mqsR_4. For both diseases, the transgene expression had a significantly negative correlation with AUDPC (r = − 0.5346, *P* < 0.05, for citrus canker and r = − 0.6129, *P* < 0.05, for CVC), indicating that the higher the gene expression, the slower the progress of the diseases. The mechanism by which the transgenic plants reduce bacterial population and symptoms are not fully understood.

The *Citrus* transgenic lines do not show any sort of developmental or morphological alterations (Fig. S5). Curiously, phytotoxicity was observed in *Arabidopsis thaliana* expressing the *yoeB* from *Streptococcus pneumoniae*^44^ and *Nicotiana tabacum* expressing *mazF* from *Escherichia coli*^45^. We believe that these differences are related to the nature of the toxins because tobacco plants expressing MqsR also showed no alterations in phenotype, similar to the citrus plants (Figure S5). Therefore, the use of MqsR is very promising because it did not induce developmental or morphological alterations for different plant species. These results are particularly important given that bacteria are becoming resistant to antimicrobial compounds, such as *X. citri* to copper^4^, the main compound used to prevent citrus canker, via phenomena not restricted to this phytopathogen^46–49^. This study opens new perspectives for exploring the use a toxin from TA systems to develop novel technology with the potential to control plant bacterial diseases.

## Methods

### Detection of MqsR in *X. citri* cells

*X. fastidiosa* MqsR protein was obtained as in Merfa and colleagues^14^. *X. citri* was cultivated in NBY broth. Two different inoculum concentrations were used (OD_600_ 0.5 and 1.0) with and without 100 µg/mL of MqsR. All samples were cultivated for 2 h at 28 °C at 150 rpm. Subsequently, each sample was divided in two and centrifuged (10,000×*g* for 1 min), and the bacteria were subjected to different treatments. In the first (non-washed), the pellets were resuspended in 90 µL of sample buffer (62.5 mM Tris–HCl, pH 6.8, 20% glycerol (v/v), 2% SDS (w/v), 5% β-mercaptoethanol (v/v)), kept at 95 °C for 5 min, and centrifuged at 11,000×*g* for 10 min. An aliquot of 10 µL was subjected to 15% SDS-PAGE. In the second treatment (washed), the cells were washed with 500 µL of deionized water, centrifuged and resuspended with sample buffer. After SDS-PAGE, the proteins were transferred to a nitrocellulose membrane using the Multiphor II Novablot (GE Healthcare). The Snap i.d. 2.0. (EMD Millipore, Billerica, MA, USA) system was used for blocking the membrane with bovine serum albumin at 1% (w/v). The anti-MqsR primary antibody^14^ was used at a dilution of 1:1000, and the anti-rabbit secondary antibody coupled to horseradish peroxidase (HRP) (Promega) at a dilution of 1:5,000 in combination with the Amersham ECL Western Blotting Detection Reagent (GE Healthcare). Chemiluminescence was detected by exposure to Amersham Hyperfilm MP (GE Healthcare). Two gels were run in parallel; one was stained with coomassie blue for protein loading visualization and the other was used for Western blot.

For the toxin degradation assay total RNA from *X. citri* was extracted using RNeasy^®^ Plus Minikit (Qiagen). Afterward, 100 µg/mL of MqsR protein was incubated with 1 µg of total RNA for 10 min at RT. The treated RNA was visualized in 1% agarose gel stained with ethidium bromide.

### Growth curve of *X. citri* in the presence of MqsR

*X. citri* was cultivated overnight in NBY broth, diluted to an optical density (OD_600_) of 0.01 and distributed into a 96-well culture plate (200 µL/well). Concentrations of 25, 50, 100, and 200 µg/mL of purified MqsR were added to *X. citri* suspensions, and for the control the MqsR elution buffer without the toxin was used. The plates were incubated at 28 °C for 48 h with 10 s intervals of shaking at 120 rpm and 10 s stop intervals using Varioskan Flash (Thermo-Fisher). OD_600_ measurements were obtained every hour. The data was used for statistical analysis using *t*-Student (*P* < 0.05).

### Construction of mqsA and *X. citri* transformation

The bacterial strains and plasmids used to construct a *X. citri* transformant overexpressing MqsA are listed in Table S2. It was used the pBBr1MCS2 vector^50^ for cloning *mqsA* (XF2491; http://www.lbi.ic.unicamp.br/xf/) using the Phusion protocol (Thermo Fischer Scientific) to generate pBBr1MCS2-mqsA. The *X. citri* strain 306 was transformed with pBBr1MCS2-*mqsA* by electroporation (2 kV, 25µF, 200 Ω).The transformant cells were selected on NBY plates (0.5% peptone, 0.3% meat extract, 0.2% yeast extract, 0.2% K_2_HPO_4_, 0.05% KH_2_PO_4_) containing 50 μg/mL of kanamycin, and the transformation was confirmed by PCR using specific primers for *mqsA* (MqsA(pBBr)F-SalI—TAT GTC GAC CCC ATT CCT GCG GAG TGC CCC ATG AGA TGT CCA TGC GGC and MqsA(pBBr)R-XbaI—TGC GTC TAG ATT AGA AAC TCT TCA CTT CGT TGA GC). The expression of the MqsA was confirmed by Western blot using a polyclonal antibody previously developed^14^.

### Construction of mqsR vectors and *A. tumefaciens* transformation

The DNA sequence of *mqsR* from the 9a5c strain of *X. fastidiosa* fused to the signal peptide (SP) sequence of *attacin A* from *Triclopusia ni*, which is known for directing proteins to the extracellular environment^29^, was synthetized by Integrated DNA Technology^®^. This sequence was cloned into the pRT101 vector under the control of the 35S-RNA promoter with a poly-adenine tail of *Cauliflower mosaic virus* using *XhoI* and *EcoRI* restriction sites. The resultant cassette was digested with *PstI* and cloned into pCambia2301, generating SP*mqsR-*pCambia2301.

### Citrus transformation and selection

Seeds of “Carrizo” citrange (*C. sinensis* (L.) Osb. x *Poncirus trifoliata* (L.) Raf.) and “Pineapple” sweet orange (*C. sinensis* (L.) Osbeck) were germinated in half-strength MS medium^34^. Transformation of epicotyls was performed using *A. tumefaciens* strain EHA 105 as described by Caserta and colleagues^34^.

For GUS activity test, a piece of leaf was excised and incubated in 100 µL of phosphate buffer containing 5-bromo-4-chloro-3-indolyl-β-D-glucuronide for 16 h at 37 °C. Shoots showing blue staining were considered positive for genetic transformation. In addition, the plants were analysed by PCR using total DNA as template with specific primers for 35S-forward (GAATTCAACATGGTGGAGCACGACAC) and *mqsR*-reverse (GACGTGAGCGCCAAGACAA) to confirm the presence of the target gene. Positive plants were grafted onto Rangpur lime rootstock and moved to a greenhouse.

### mqsR expression in citrus transgenic lines

Total RNA was extracted from leaves of each transgenic line using the Tri reagent® (Qiagen) and treated with RNase-Free DNase (Qiagen). A total of 1 µg of RNA from each sample was used to synthesize cDNA with oligo-dT following the protocol of the GoScript^™^ Reverse Transcription System (Promega). These samples were used for real time quantitative PCR (qRT-PCR) assays with a GoTaq^®^ DNA Polymerase kit (Promega) and 5 pmol of each specific primer for *mqsR* (forward, 5′-CTGGCAAGGTCAGGGCTACA-3′, and reverse, 5′-GACGTGAGCGCCAAGACAA-3′). The cyclophilin gene was used as endogenous control (forward, 5′-AGAGTATGCAGAGGAATGG-3’, and reverse, 5′-GTCCTTAACAGAAGTCCGT-3′)^31^. The primers efficiency was calculated in MINER^51^. Each qRT-PCR was performed in duplicate using an ABI 7500 System (Applied Biosystems). The results were analysed using the relative quantification method^52^.

Estimation of the transgene copy number was done as previously described^31,53,54^, by comparing the amplification of the transgene to a control gene present in single copy in the genome of citrus (LTP, id NM_001288873)^55^. For this, a single amplification using plant DNA was performed using specific primers for 35S-*mqsR* (35S F 5′-GACGCACAATCCCACTATCC-3′ and *mqsR* R 5′-GACGTGAGCGCCAAGACAA-3′) and the LTP (LTP F 5′-GCTGCCGCCAGAACCA-3′ and LTP R 5′-GCGGCTTGCTTCAAGCA-3′). The amplification reactions were performed using the GoTaq^®^ qPCR Master Mix (Promega) as described above, using the ABI 7500 system (Applied Biosystems). The estimated copy number was calculated as described^53^.

### Confirmation of MqsR protein expression in citrus transgenic lines

Four leaf discs from the Pineapple (Pi_*mqsR_1, 2, 3* and *4*) and Carrizo (C_*mqsR_1*) transgenic lines and respective wild-type were used for protein extraction^56^. Western blot was carried by transferring the proteins to a *Hybond-C* nitrocellulose membrane using a Trans-Blot Turbo System (Bio-Rad). The membrane was incubated in 300 mL of blocking solution (PBS buffer, 0.1% Tween 20, 1% bovine serum albumin) overnight. The Snap i.d. 2.0. (EMD Millipore) system was used for treatments with the anti-MqsR primary antibody at a dilution of 1:250 and the anti-rabbit secondary antibody-HRP (Promega) at a dilution of 1:5,000. The protein was detected in a ChemiDoc™ XRS + System (Bio-Rad) using the Amersham ECL Western Blotting Detection Reagent.

The tissue-print analysis was also used for protein detection^57^. Wild-type plants were blotted in nitrocellulose membrane for preadsorption of the primary antibody. The membrane was incubated overnight in blocking solution followed by an incubation of 5 h shaking with anti-MqsR at a dilution of 1:250. Another membrane with printing samples from two different stems for each transgenic line, as well as wild-type as negative control, and the purified MqsR protein as positive control, was incubated overnight in blocking solution. The membranes were incubated with the primary antibody obtained after preadsorption for 1 h, washed and incubated for 15 min with anti-rabbit secondary antibody conjugated with HRP (1:5,000) (Promega). MqsR protein was detected with the Amersham ECL Western Blotting Detection Reagent after exposure to ChemiDocTM (Bio-Rad). Dot blot was performed with the same protein extraction protocol described above^56^ with some modifications. Coomassie brilliant blue was not added to the β-buffer. The membrane was plotted with 5µL drops in eight spots for each sample. The same protocol described for tissue-print was performed in dot blot. The dot blot was analysed using the volume tool from Image Lab Software (Bio-Rad). The same area was quantified for each sample and the differences were analysed by Student *t* test.

### Evaluation of symptoms and detection of MqsR in *X. citri* cells

Detached leaves from transgenic and wild-type plants were infiltrated with *X. citri* constitutively expressing GFP^58^ (referred to as *X*. *citri*_GFP). The inoculum was standardized to an OD_600_ of 0.001 (10^4^ CFU/mL). An aliquot of 100 µL was infiltrated on Pineapple sweet orange and Carrizo leaves and incubated for 14 days at 28 °C. Three leaves at the same age were used for each experiment, and five independent experiments were performed. The symptoms were evaluated 7 and 14 DAI. Fluorescence analysis of the lesions was performed using an Olympus MVX10 (U-MGFPHQ filter) microscope.

The infiltrated leaves were also used to assess the presence of the MqsR protein in *X. citri*. At 14 DAI, the leaves were cut and submerged in PBS buffer for 15 min to extract the *X. citri* cells from the plant tissues. The cells were collected, resuspended in sample buffer, lysed by heating and loaded on 15% SDS-PAGE. Immunoblotting was performed as described above with the anti-MqsR, anti-GFP, and the HRP-conjugated anti-Rabbit antibodies diluted 1:500, 1:2000, and 1:5000 respectively.

### Evaluation of citrus canker and CVC symptoms under greenhouse conditions

Ten buds of each transgenic line and wild-type were grafted onto *Citrus limonia* Osbeck. To avoid chimeric clones, leaves from all of the grafted transgenic lines were previously screened for GUS activity. For citrus canker evaluation, five non chimeric transgenic lines and the wild-type were sprayed with *X. citri* at 10^8^ CFU/mL. After symptoms appearance, each plant was scored weekly for 7 weeks by three different evaluators using a diagrammatic scale^32^.

For CVC, Carrizo was not used since only sweet oranges are susceptible to *X. fastidiosa*^59^. For Pinneaple transgenic lines, at least 10 clones were inoculated with 20 µL of a suspension with 10^7^ cells/mL of the 9a5c strain of *X. fastidiosa*. DNA of these plants was isolated 45 DAI and used to detect the presence of *X. fastidiosa* by PCR^60^. A total of six PCR positive plants were used per line in the subsequent analysis. The severity was scored by three different evaluators using a diagrammatic scale^33^ every 30 days for 4 months after the symptoms appearance. Symptom severity along the disease development was calculated as the area under the disease-progress curve (AUDPC) using the trapezoidal integration method^61^ and analysed by *t*-Student (*P* < 0.05).

## Supplementary Information

Supplementary Information 1.

Supplementary Information 2.

Supplementary Information 3.

## Data Availability

The data that support the findings of this study are available from the corresponding author upon reasonable request.
