# Supplementary material for: MqsR toxin as a biotechnological tool for plant pathogen bacterial control
Source: Sci Rep. 2022 Feb 18;12:2794. doi: 10.1038/s41598-022-06690-x (PMC8857320; doi:10.1038/s41598-022-06690-x)
Supplement: Supplementary file 1 — Supplementary Information 1. [file 41598_2022_6690_MOESM1_ESM.pdf]

MqsR toxin as a biotechnological tool for plant pathogen bacterial control

Reinaldo Rodrigues de Souza-Neto[1,2], Isis Gabriela Barbosa Carvalho[1], Paula Maria Moreira Martins[1], Simone Cristina Picchi[1], Juarez Pires Tomaz[3], Raquel Caserta[1], Marco Aurélio Takita[1] and Alessandra Alves de Souza[4]

[1]Centro de Citricultura "Sylvio Moreira", Instituto Agronômico de Campinas, Cordeirópolis, SP, Brazil.

[2]Departamento de Genética, Evolução e Bioagentes, Instituto de Biologia, Universidade Estadual de Campinas, SP, Brasil.

[3]Instituto Agronômico do Paraná, Londrina, PR, Brazil.

[4]Centro de Citricultura "Sylvio Moreira", Instituto Agronômico de Campinas, Cordeirópolis, SP, Brazil. desouza@ccsm.br

Methods

Isolation of *X. citri* population

The bacterial population in the leaves was assessed at 14 DAI by plating serial dilutions as described by Caserta et al. (2014). Briefly, leaf discs 1 cm in diameter were cut around the inoculation point and homogenized in phosphate-buffered saline (PBS). The samples were diluted, plated on NBY medium and incubated at 28° C for 48 h. The CFUs were counted, and the bacterial population of each leaf from the transgenic lines was compared with the non-transgenic leaves. The results were used for statistical analysis using *t*-Student.

Gene expression of *X. citri* genes when infiltrated in transgenic leaves

The same procedure performed for isolation of *X. citri* was also performed for bacterial gene expression experiments; however, prior to RNA extraction, leaf discs were frozen in liquid nitrogen. Then, RNA extraction was performed using RNeasy® Plus Minikit (Qiagen Inc., Valencia, CA, USA), followed by treatment with RNase-Free DNase (Qiagen Inc., Valencia, CA, USA) to avoid DNA contamination. cDNA was synthesized using 1 µg of each sample and random primers as described in the GoScriptTM Reverse Transcription System (Promega Corporation, Madison, WI, USA). qPCR was performed using a GoTaq® DNA Polymerase kit (Promega Corporation, Madison, WI, USA) with 5 pmol of each primer for the fliC (forward, 5'–TCAAGCAGTTGACCTCTGAAATC-3', and reverse, 5'-GCGCCGACCTGGAACAG-3') (Caserta et al., 2014) and rpoD (forward, 5'-GGATCCGTCAGGCCATCAC-3', and reverse, 5'-GCGCCGACCTGGAACAG-3') genes. As an internal control, 16S ribosomal RNA primers (forward, 5'-CCGGATTGGAGTCTGCAACT-3', and reverse, 5'–ACGTATTCACCGCAGCAAT-3') were used for both samples (Caserta et al., 2014). qRT-PCR was performed in duplicate using an ABI 7500 System (Applied Biosystems, Foster City, CA, USA). The results were analysed with the 7500 System software using the relative quantification method (Livak and Schmittgen, 2001). t-Student ($P < 0.05$) analysis was used to verify differences between the transgenic and wild-type plants.

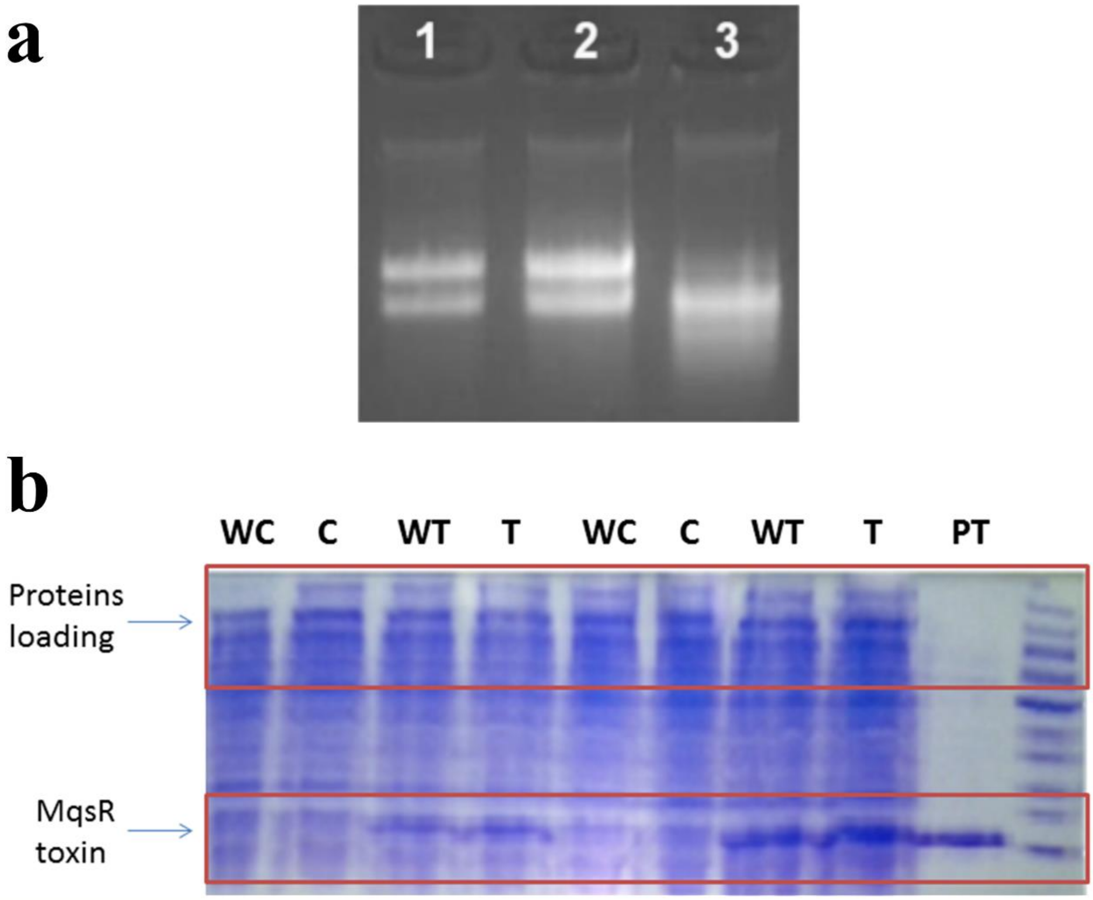

Figure S1 MqsR analysis. (a) Assay of *X. ctri* RNA with addition of 100 µg/mL. 1 – pure RNA; 2 – RNA in 50 mM phosphate buffer with 75 mM of imidazole (mock); 3 – RNA and MqsR in 50 mM phosphate buffer with 75 mM of imidazole. (b) SDS-PAGE total protein for loading control of MqsR immunoblotting detection with *X. citri* cells. WC – washed *X. citri* control; C – non-washed *X. citri* control; WT – washed *X. citri* treated with MqsR; and T – non-washed treated with MqsR. PT is purified MqsR (positive control). Protein loading – Equivalent proteins loading. MqsR toxin – expected size of MqsR.

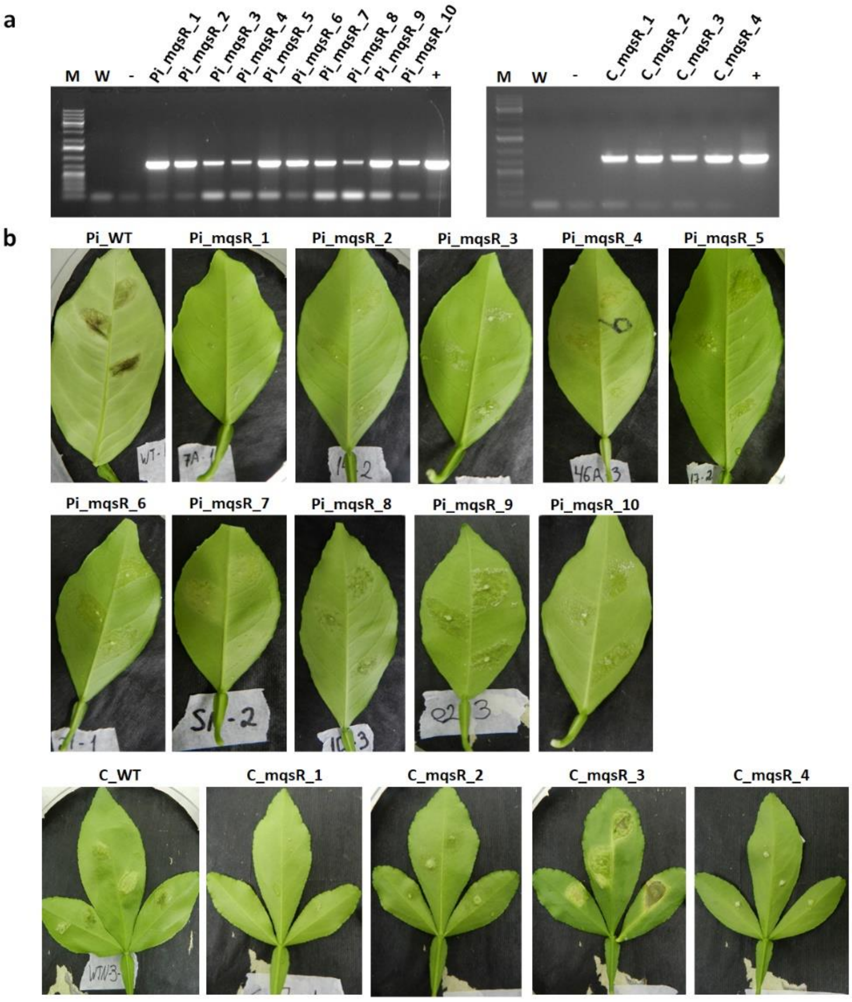

Figure S2 Screening of transgenic lines. (a) *mqsR* amplification (700 bp, 35S promoter + mqsR). M, O'Gene Ruler Plus (Thermo Fisher Scientific); C -, negative control (water); C +, positive control (SPmqsR-pCambia2301); Pi_WT, wild-type Pineapple sweet orange; Pi_mqsR_1, 2, 3, 4, 5, 6, 7, 8, 9, and 10. Pineapple transgenic lines transformed with mqsR; C_WT, wild-type Carrizo; C_mqsR_1, 2, 3, and 4. Carrizo transgenic line transformed with mqsR. (b) Screening of transgenic lines at 14 DAI with *X. citri*-GFP. The name of each line is shown on top of each image.

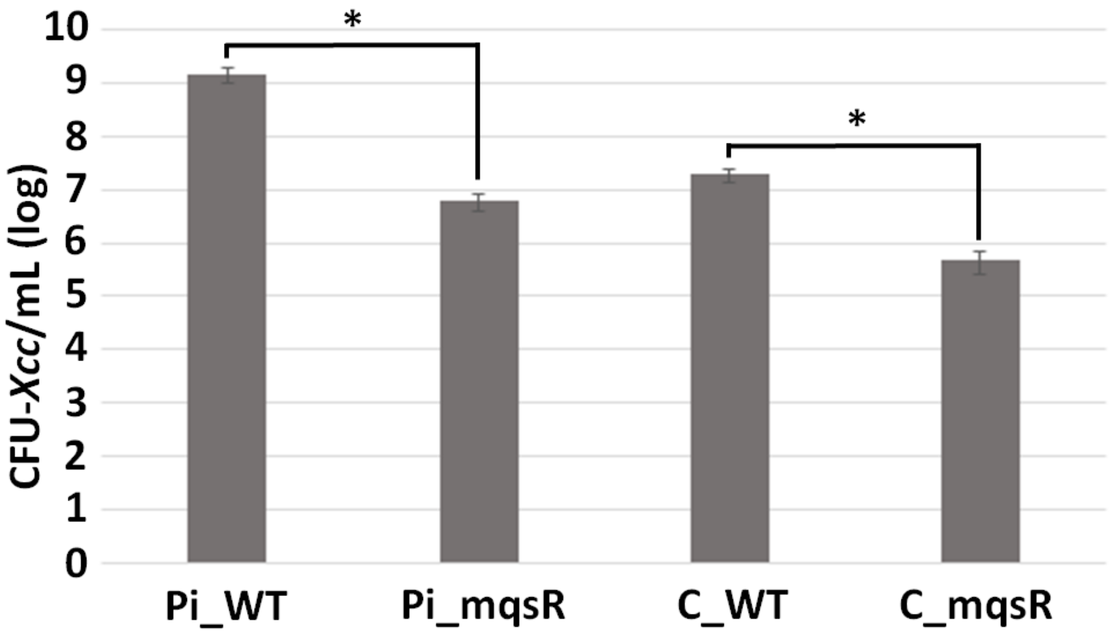

Figure S3 Bacterial population of *X. citri* isolated from infiltrated leaves. Pi_WT, wild-type Pineapple sweet orange; Pi_mqsR, transgenic Pineapple sweet orange (average from all three lines); C_WT, wild-type Carrizo; and C_mqsR, transgenic Carrizo. The results are the means of three replicates. Bars indicate the standard error of the means. Asterisks show statistically significant differences analysed by t-Student tests ($P < 0.05$).

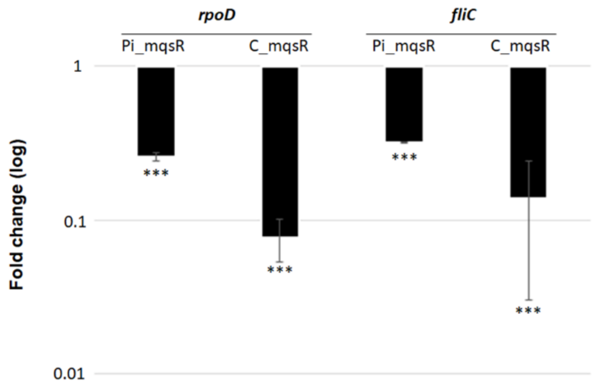

Figure S4 Relative quantification of *rpoD* and *fliC* in *X. citri* from transgenic lines. Gene expression was evaluated for bacteria from Pi_mqsR and C_mqsR in relation to those from their respective wild-type plants at 14 DAI. The results are the means of three independent experiments. Bars indicate the standard error of the means; *** indicates

significant differences ($P < 0.001$) between the mean values obtained for each gene compared with the non-transformed plants.

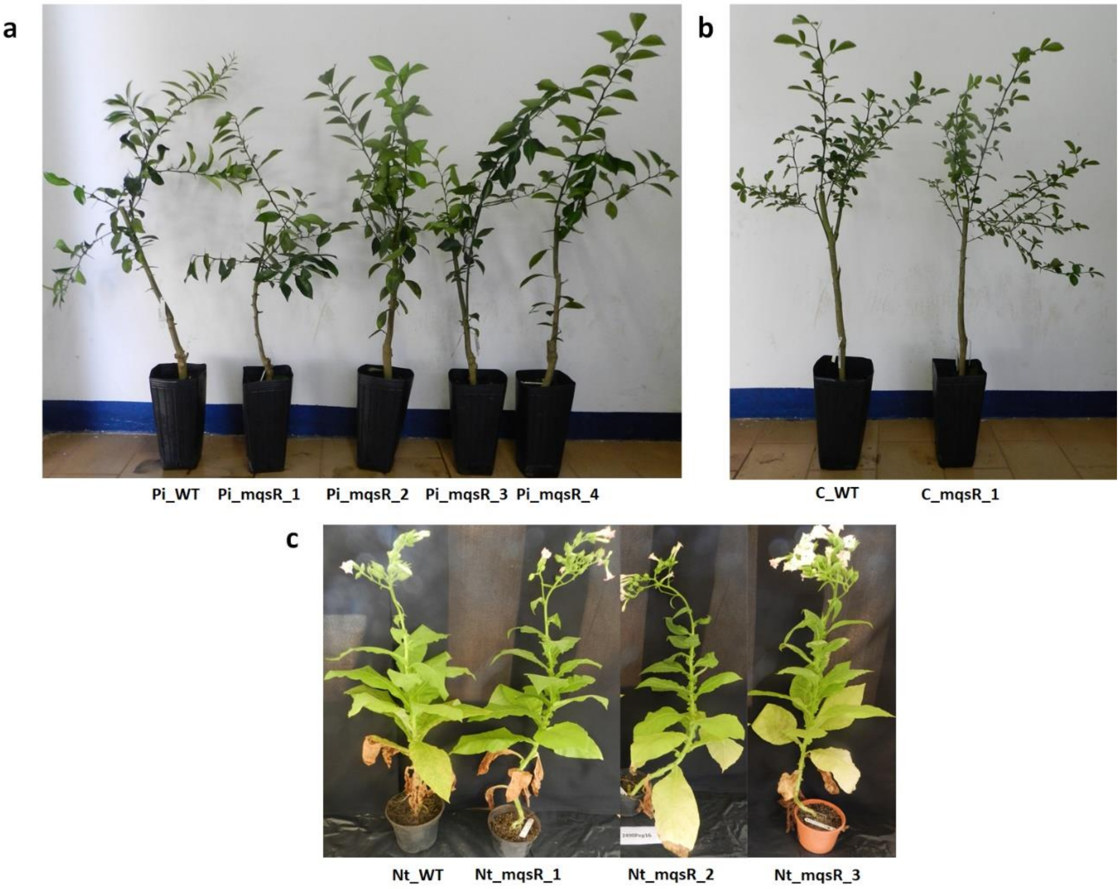

Figure S5 MqsR transgenic plants growing in a greenhouse. (a and b) Three-year-old citrus transgenic plants showing no visible phenotypic differences compared with their respective wild-type plants. Pi_WT, wild-type Pineapple sweet orange type; Pi_mqsR_1, 2, 3 and 4, Pineapple transgenic lines transformed with *mqsR*; C_WT, wild-type Carrizo; and C_mqsR_1, Carrizo transgenic line transformed with *mqsR*. (c) No detectable phenotypic differences were observed in *Nicotiana tabacum* expressing MqsR compared with the wild-type at 70 days after germination. Nt_WT, wild-type *N. tabacum*, and Nt_mqsR_1, 2, and 3, correspond to different *N. tabacum* transgenic lines.
