# Supplementary material for: MqsR toxin as a biotechnological tool for plant pathogen bacterial control
Source: Sci Rep. 2022 Feb 18;12:2794. doi: 10.1038/s41598-022-06690-x (PMC8857320; doi:10.1038/s41598-022-06690-x)
Supplement: Supplementary file 2 — Supplementary Information 2. [file 41598_2022_6690_MOESM2_ESM.pdf]

MqsR toxin as a biotechnological tool for plant pathogen bacterial control

Reinaldo Rodrigues de Souza-Neto[1,2], Isis Gabriela Barbosa Carvalho[1], Paula Maria Moreira Martins[1], Simone Cristina Picchi[1], Juarez Pires Tomaz[3], Raquel Caserta[1], Marco Aurélio Takita[1] and Alessandra Alves de Souza[4]

[1]Centro de Citricultura "Sylvio Moreira", Instituto Agronômico de Campinas, Cordeirópolis, SP, Brazil.

[2]Departamento de Genética, Evolução e Bioagentes, Instituto de Biologia, Universidade Estadual de Campinas, SP, Brasil.

[3]Instituto Agronômico do Paraná, Londrina, PR, Brazil.

[4]Centro de Citricultura "Sylvio Moreira", Instituto Agronômico de Campinas, Cordeirópolis, SP, Brazil. desouza@ccsm.br

Table S1. Estimated copy number of the *mqsR* gene in the citrus transgenic lines.

Transgenic Line	*Ct gene of interest (*mqsR*)	Ct gene of control (*LTP*)	Ratio	Estimated copy number
**Pi_mqsR_1**	21.67464	19.81128	0.902587	1
**Pi_mqsR_2**	22.58349	20.013	0.902759	1
**Pi_mqsR_3**	22.10529	20.56012	0.909199	1
**Pi_mqsR_4**	19.88684	19.8468	0.907901	1
**C_mqsR_1**	20.44237	20.90364	0.916722	1
**Efficiency of primers**	0.817932	0.899307	-	-
