# Supplementary material for: MqsR toxin as a biotechnological tool for plant pathogen bacterial control
Source: Sci Rep. 2022 Feb 18;12:2794. doi: 10.1038/s41598-022-06690-x (PMC8857320; doi:10.1038/s41598-022-06690-x)
Supplement: Supplementary file 3 — Supplementary Information 3. [file 41598_2022_6690_MOESM3_ESM.pdf]

MqsR toxin as a biotechnological tool for plant pathogen bacterial control

Reinaldo Rodrigues de Souza-Neto[1,2], Isis Gabriela Barbosa Carvalho[1], Paula Maria Moreira Martins[1], Simone Cristina Picchi[1], Juarez Pires Tomaz[3], Raquel Caserta[1], Marco Aurélio Takita[1] and Alessandra Alves de Souza[4]

[1]Centro de Citricultura "Sylvio Moreira", Instituto Agronômico de Campinas, Cordeirópolis, SP, Brazil.

[2]Departamento de Genética, Evolução e Bioagentes, Instituto de Biologia, Universidade Estadual de Campinas, SP, Brasil.

[3]Instituto Agronômico do Paraná, Londrina, PR, Brazil.

[4]Centro de Citricultura "Sylvio Moreira", Instituto Agronômico de Campinas, Cordeirópolis, SP, Brazil. desouza@ccsm.br

Table S2. Bacterial strains and plasmids used in this study.

**Strains and plasmids**	**Genotype/relevant characteristics**	**Source**
Xanthomonas citri subsp. citri		
Strain 306	Wild-type	da Silva et al., 2002
306-*mqsA*	306 strain bearing the pBBr1MCS2-*mqsA* plasmid	this study
306-pBBr1MCS2	306 strain bearing the pBBr1MCS2	this study
E. coli		
DH10B	F- mcrA Δ(mrr-hsdRMS-mcrBC) φ80lacZΔM15 ΔlacX74 recA1 endA1 araD139 Δ (ara, leu)7697 galU galK λ- rpsL nupG /pMON14272 / pMON7124	Invitrogen
**Plasmids**		
pBBr1MCS2	Km^R, cloning vector	Kovach et al., 1995
pBBr1MCS2-*mqsA*	Km^R	this study
